# A photonic frequency discriminator based on a two wavelength delayed self-heterodyne interferometer for low phase noise tunable micro/mm wave synthesis

**DOI:** 10.1038/s41598-018-31712-y

**Published:** 2018-09-12

**Authors:** N. Kuse, M. E. Fermann

**Affiliations:** 1 IMRA America Inc., Boulder Research Labs, 1551 South Sunset St, Suite C, Longmont, CO 80501 USA; 2 IMRA America Inc., 1044 Woodridge Ave, Ann Arbor, MI 48105 USA

## Abstract

Low phase noise frequency synthesizers are of paramount interest in many areas of micro-mm wave technology, encompassing for example advanced wireless communication, radar, radio-astronomy, and precision instrumentation. Although this broad research field is not bereft of methods for the generation of either low phase noise micro- or mm waves, no universal system applicable to low phase noise generation for micro and mm waves has yet been demonstrated. Here we propose a new photonic frequency discriminator based on a two wavelength delayed self-heterodyne interferometer which is compatible with such an objective. The photonic frequency discriminator can be a reference both for micro and mm waves to lower their phase noise. As a proof-of-concept, we demonstrate a low phase noise tunable OEO (6–18 GHz) and locking of a heterodyne beat between two cw lasers (10–400 GHz) with low relative phase noise. The required components for the photonic frequency discriminator are off-the-shelf and can be readily assembled. We believe this new type of photonic frequency discriminator will enable a new generation of universal precision tunable sources for the X, K, V, W and mm-bands and beyond.

## Introduction

Low phase noise micro – mm wave oscillators are indispensable for wireless communication^1,2^, radar^3^, radio-astronomy^4^, and modern instrumentation. For example, low phase noise mm waves facilitate the use of complex modulation formats for mm wave communication systems, which have been suggested for overcoming the capacity bottleneck for future wireless ultra-high bandwidth data transfer. Another example is Doppler radar, in which sensitivity is strongly affected by phase noise. Traditionally low phase noise frequency synthesis is performed via frequency multiplication (×N) of low phase noise microwave reference oscillators. While this scheme allows for the generation of frequencies of several hundred GHz, the high carrier frequency phase noise is inevitably magnified by N^2^ in power spectral density (PSD) compared to the reference oscillator. Moreover, excess noise typically leads to a degradation of phase noise far in excess of N^2^. To overcome this, photonic methods have been established that promise to go beyond the phase noise limits of classical microwave technology. One such method is optical frequency division (OFD), i.e. frequency division from optical to microwave frequency ranges, through fully-stabilized optical frequency combs^5–8^. However, OFD requires a sophisticated optical reference^9^ as well as fully-stabilized mode-locked lasers^10^. Both are hard to operate, complex, bulky and expensive; therefore the use of OFD has been confined to specialized metrology labs. Moreover, the best performance is available only at a fixed carrier frequency. Furthermore, high carrier frequency generation beyond 100 GHz is very challenging with frequency combs. There are two alternative photonic methods, which can generate tunable carrier frequencies and have been used for real world application: opto-electronic oscillators (OEOs) (<50 GHz)^11–13^ and heterodyning of two cw lasers (>50 GHz)^14–18^. In principle, the achievable phase noise with both methods does not depend on carrier frequency.

OEOs comprise a loop with optic – electro and electro – optic conversion and oscillate at frequencies corresponding to integer multiples of the free-spectral range (FSR) of the loop. To select a particular oscillation mode, an RF bandpass filter is installed in the loop. RF bandpass filters can be tunable by using photonic RF filters^19–21^ or tunable RF filters^22,23^, enabling tunable OEOs^19–23^. Because both photonic RF filters and tunable RF filters are widely tunable, OEOs can be designed to oscillate in a large frequency range. For example, ref.^20^ reported oscillation frequencies from DC to 60 GHz by using a photonic RF filter based on phase shifted fiber Bragg gratings. However, to date, the phase noise of tunable OEOs is not truly competitive with what is achievable with frequency synthesizers based on conventional microwave technology (summarized in the discussion section).

Although tunable OEOs exhibit excellent tunability without phase noise degradation, inducing oscillation around the W band (75–110 GHz) or beyond is very difficult because of the requirement for high bandwidth RF components. At a certain carrier frequency, the method based on heterodyning two cw lasers becomes more powerful than OEOs. Heterodyning at a photo detector generates micro/mm waves with a carrier frequency equal to the frequency separation between the two cw lasers. By changing the optical frequency of one of the two cw lasers, the generated carrier frequency can be widely tuned. However, the phase noise of the micro/mm waves is governed by relative phase noise of the two cw lasers; which can be very high if the two lasers are independent. It is therefore desirable to provide a strong level of correlation between the two cw lasers to reduce their relative phase noise. Appropriate laser correlation can be realized through optical frequency combs, including both mode-locked optical frequency combs^14^ and electro-optic combs (EO combs)^15,16^, or having the lasers share a common optical cavity^17,18^.

In this letter, we introduce a novel photonic frequency discriminator (PFD), which is used to reduce the phase noise both for tunable OEOs and heterodyning of two cw lasers. In a proof-of-concept demonstration, a low phase noise tunable OEO (6–18 GHz) and a low phase noise tunable heterodyne beat from two cw lasers (10–400 GHz) are shown via locking to the PFD.

## Working Principle

**Figure 1:**
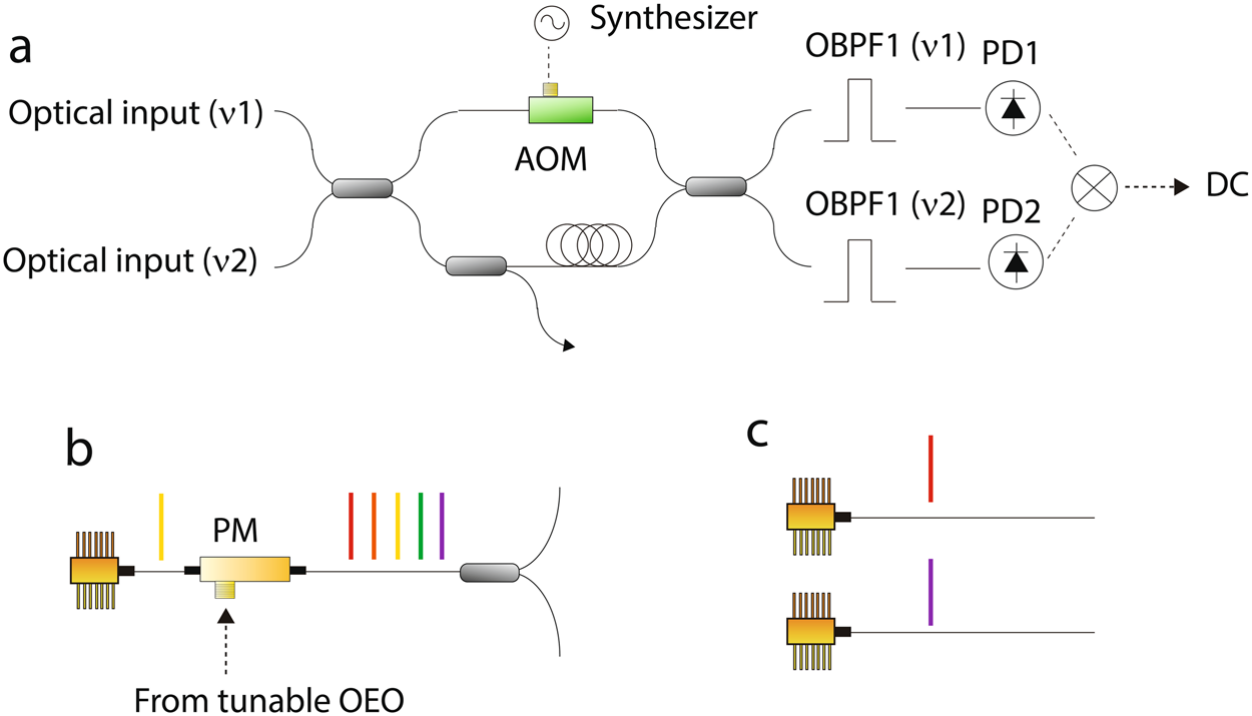
(**a**) Schematic of the photonic frequency discriminator. AOM, acousto-optic modulator; OBPF, optical bandpass filter; PD, photo detector. (**b**) Schematic of two optical inputs for phase noise reduction of a tunable OEO. (**c**) Schematic of two optical inputs for heterodyning of two cw lasers.

The PFD is based on a two wavelength delayed self-heterodyne interferometer (TWDI)^24–26^ as shown in Fig. 1a. The TWDI has two different wavelength optical inputs and a DC output. The DC output of the PFD contains the relative phase noise between the two optical inputs with a delayed transfer function^27^ (H(jf), please refer to the supplementary material) of an imbalanced Mach-Zehnder interferometer (iMZI). The two optical inputs with a phase noise PSD of L_in1_(f) and L_in2_(f) are coupled into the iMZI through a 2 × 2 optical coupler. One arm in the iMZI has a long fiber delay (~200 m for a tunable OEO and 50 m for heterodyning two cw lasers) and the other arm has an optical frequency shifter (f_AOM_ ~ 160 MHz) in the form of an acousto-optic modulator (AOM). After combing the light from the two iMZI arms through an optical coupler, the two outputs from the optical coupler are optically bandpass filtered to separate the two optical inputs. At the photo detectors, signals at the AOM frequency for each optical input are generated. Mathematically, the photocurrents (i_1(2)_(t)) after the PDs in the time domain can be expressed as,

1 $${i}_{1(2)}(t)\propto cos(2\pi {f}_{AOM}t+2\pi {\nu }_{in1(2)}\tau +{\phi }_{AOM}(t)+{\phi }_{in1(2)}(t)-{\phi }_{in1(2)}(t-\tau )).$$

Here, ν_in1(2)_, φ_AOM_(t), φ_in1(2)_(t), and τ are the optical frequency of input 1(2), phase noise of the synthesizer for the AOM, phase noise of input 1(2), and delay time in the iMZI. By mixing these two signals, the output from the mixer (V_mix_(t)) is

2 $${V}_{mix}(t)\propto cos(2\pi ({\nu }_{in1}-{\nu }_{in2})\tau +({\phi }_{in1}(t)-{\phi }_{in1}(t-\tau ))-({\phi }_{in2}(t)-{\phi }_{in2}(t-\tau ))).$$

Note that common noise is cancelled out through the mixing process, resulting in no detrimental effect from the AOM and down-conversion of fiber delay noise (i.e. fluctuations of τ) in the iMZI from ν_in1_ (or ν_in2_) to ν_in1_ − ν_in2_. The phase noise PSD of the signal after the the mixer (L_mix_(f)) can be evaluated at quadrature as

3 $${L}_{mix}(f)={|H(jf)|}^{2}({L}_{in1}(f)-{L}_{in2}(f)\,).$$

L_mix_(f) is used as an error signal, and a feedback loop is configured to make L_mix_(f) as small as possible.

In the case of a tunable OEO, the two iMZI inputs are generated via phase modulation of a single longitudinal mode cw laser via with the output from the OEO (f_OEO_) as shown in Fig. 1b, generating an EO comb. The two EO comb modes at of +/−N th order are used as the two optical inputs for the TWDI. In this case, ν_in1_ = ν_cw_ + N × f_OEO_, ν_in2_ = ν_cw_ − N × f_OEO_, φ_in1_(t) = φ_cw_(t) + N × φ_OEO_(t), and φ_in2_(t) = φ_cw_(t) − N × φ_OEO_(t) are satisfied. Here, φ_cw_(t)and φ_OEO_(t) are phase noise of the cw laser and OEO, respectively. Experimentally, all EO comb modes go through the iMZI, and the two EO comb modes (+ and − N th orders) are taken out by the two optical bandpass filters, respectively. From these relations, L_mix_(f) is

4 $${L}_{mix}(f)={(2N)}^{2}{|H(jf)|}^{2}{L}_{OEO}(f).$$

Here, L_OEO_(f) is phase noise PSD of the OEO, respectively. Note that phase noise of the cw laser is cancelled out in the mixing process^26^. By feeding back to a modulator in the OEO, using L_mix_(f) as an error signal, phase noise of the OEO is reduced. Note that although phase noise reduction of an OEO by locking to a conventional PFD based on delayed self-homodyne interferometer has been demonstrated^28^, our novel PFD based on TWDI has two significant advantages. One is higher sensitivity because of sensitivity magnification with a factor of (2N)^2^, enabled by the use of the EO comb. More importantly, the second advantage is that our PFD requires much less high bandwidth RF components. While conventional PFD detect f_OEO_ and process the signal at that frequency, our signal frequency is f_AOM_ (~160 MHz), which is much easier to process and has higher performance.

In the case of locking of two cw lasers for mm wave generation, the two optical inputs are simply the two independent cw lasers (Fig. 1c). In this case, ν_in1_ = ν_cw1_, ν_in2_ = ν_cw2_, φ_in1_(t) = φ_cw2_(t), and φ_in2_(t) = φ_cw2_(t) are satisfied. Here, ν_cw1(2)_ and φ_cw1(2)_ are optical frequency and phase noise of cw laser 1(2), respectively. From these relations, L_mix_(f) is

5 $${L}_{mix}(f)={|H(jf)|}^{2}({L}_{cw1}(f)-{L}_{cw2}(f)\,).$$

Here, L_cw1(2)_(f) is the phase noise PSD of cw laser 1(2). By feeding back to one of the two cw lasers, using L_mix_(f) as an error signal, relative phase noise between the two cw lasers is reduced. Note there are reports, in which relative phase noise between two cw lasers is reduced by locking to a fiber delay^29^. There is one significant advantage for our PFD. Previous demonstrations have detected the heterodyne signal between two cw lasers with and without fiber delay and processed the signals at that frequency, which prohibits an extension of the method to the W-band or beyond. On the other hand, in the present system, just as for the case of the OEO, the detected and processed signal frequency is the AOM frequency; therefore the frequency separation between the two cw lasers can be easily extended to the W-band or beyond.

In summary, as explained above, our novel PFD is suitable as a reference both for tunable OEOs and heterodyning of two cw lasers by just selection of appropriate optical inputs, thus presenting a unique opportunity for universal low noise frequency synthesis in the spectral range from micro – mm waves, even THz.

## Tunable OEO

**Figure 2:**
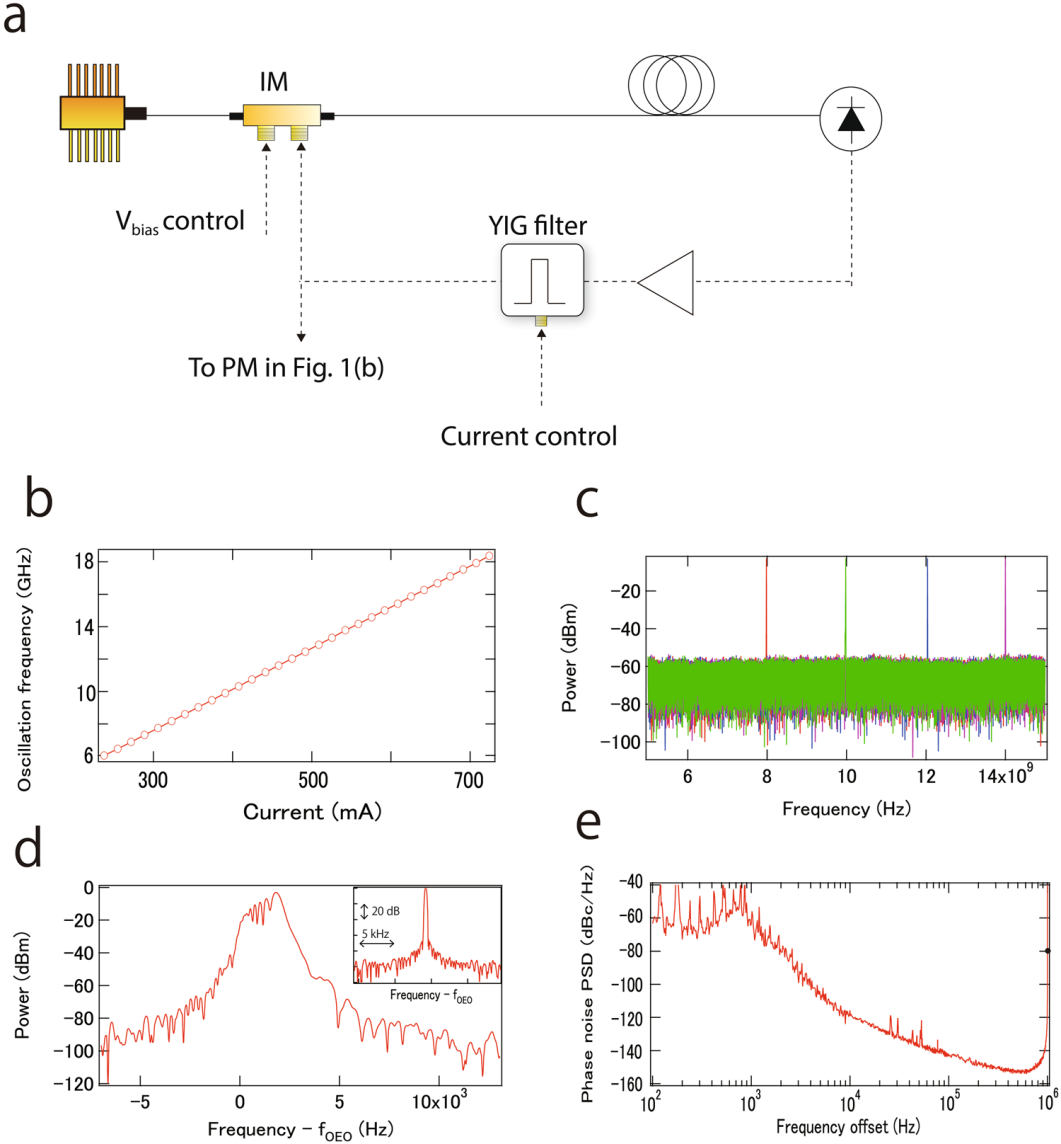
(**a**) Schematic of the tunable OEO. (**b**) OEO oscillation frequency against drive current for the YIG filter. (**c**) Coarse tuning of the OEO oscillation frequency. (**d**) Instantaneous RF spectrum of the free-running OEO measured in about 10 ms. Inset shows the RF spectrum of the OEO locked to the PFD. (**e**) Phase noise PSD of the free-running OEO for 10 GHz oscillation. Black circle at 1 MHz frequency offset is phase noise of a higher mode, showing −80 dBc. Note that if the phase noise of the spike is measured by a FFT analyzer, the phase noise looks much smaller because of the measurement resolution bandwidth, which is not correct. Many papers about OEOs do not mention this.

A schematic of the tunable OEO is shown in Fig. 2a. Tunability relies on the use of a tunable RF filter (YIG filter, 3 dB bandwidth of 40 MHz and tunability from 4 to 26.5 GHz). Although tunable OEOs have been demonstrated with YIG filters^22^, tunable OEOs with YIG filters are not a good choice for stable, low noise OEOs, because the passband of YIG filters is jittering due to the noise of the drive current, resulting in an unstable OEO oscillation frequency. However, the disadvantage can be converted to an advantage by using a YIG filter as a modulator^23,28^. In our system, the YIG filter is used not only for frequency tuning, but also as a modulator when the OEO is actively locked to the PFD. Except for the expanded use of the YIG filter, the basic OEO as presented here is constructed similarly to standard OEOs with a 200 m fiber. Please refer to the method section for more detail. Upstream of the intensity modulator, part of the RF signal is coupled out by an RF coupler, and injected to the PFD, followed by an electric amplifier. Low phase noise output can be partially coupled out after the electric amplifier. When the OEO is locked to the PNA, the bias voltage of the intensity modulator is used as a fast modulator, because the modulation bandwidth of the YIG filter is lower than that of the intensity modulator. The bias voltage of the modulator regulates the optical/RF power in the OEO loop, inducing OEO oscillation frequency modulation through OEO oscillation dynamics^28,30^. Any intensity modulation mechanism can in principle be used such as an acousto-optic modulator (AOM) or an additional intensity modulator in front of the OEO loop. Once the OEO loop gain exceeds the OEO loop loss, the OEO starts oscillating. The oscillation frequency is roughly set by the passband of the YIG filter, and determined exactly by the integer multiple of the inverse of the “effective” OEO loop delay. Note that the “effective” delay can be changed, depending on the wavelength of the cw laser^31^ or optical/RF power^28,30^. This is why the bias voltage of the intensity modulator can be used as a frequency modulator.

As shown in Fig. 2b,c, the OEO oscillation frequency can be selected from 6 to 18 GHz via changing the drive current of the YIG filter. Examples of RF spectra are shown in Fig. 2c. However, without locking, the OEO is jittering as shown in Fig. 2d (measurement time of about 10 ms). Please also refer to a supplementary movie. Figure 2e shows the free-running phase noise of the OEO. Note that to suppress the frequency jitter, the OEO is locked to the PFD with 1 kHz feedback bandwidth. The high oscillation mode frequency is pushed out to around 1 MHz because of the short OEO fiber, which is significantly shorter than for other reported tunable OEOs (summarized in the discussion section). Note that a spike at 1 MHz in Fig. 2e is an artifact from calibration, and the actual phase noise at 1 MHz is −80 dBc. By using a multi-loop configuration^32,33^, the spike can be suppressed, sacrificing ease of frequency tuning.

**Figure 3:**
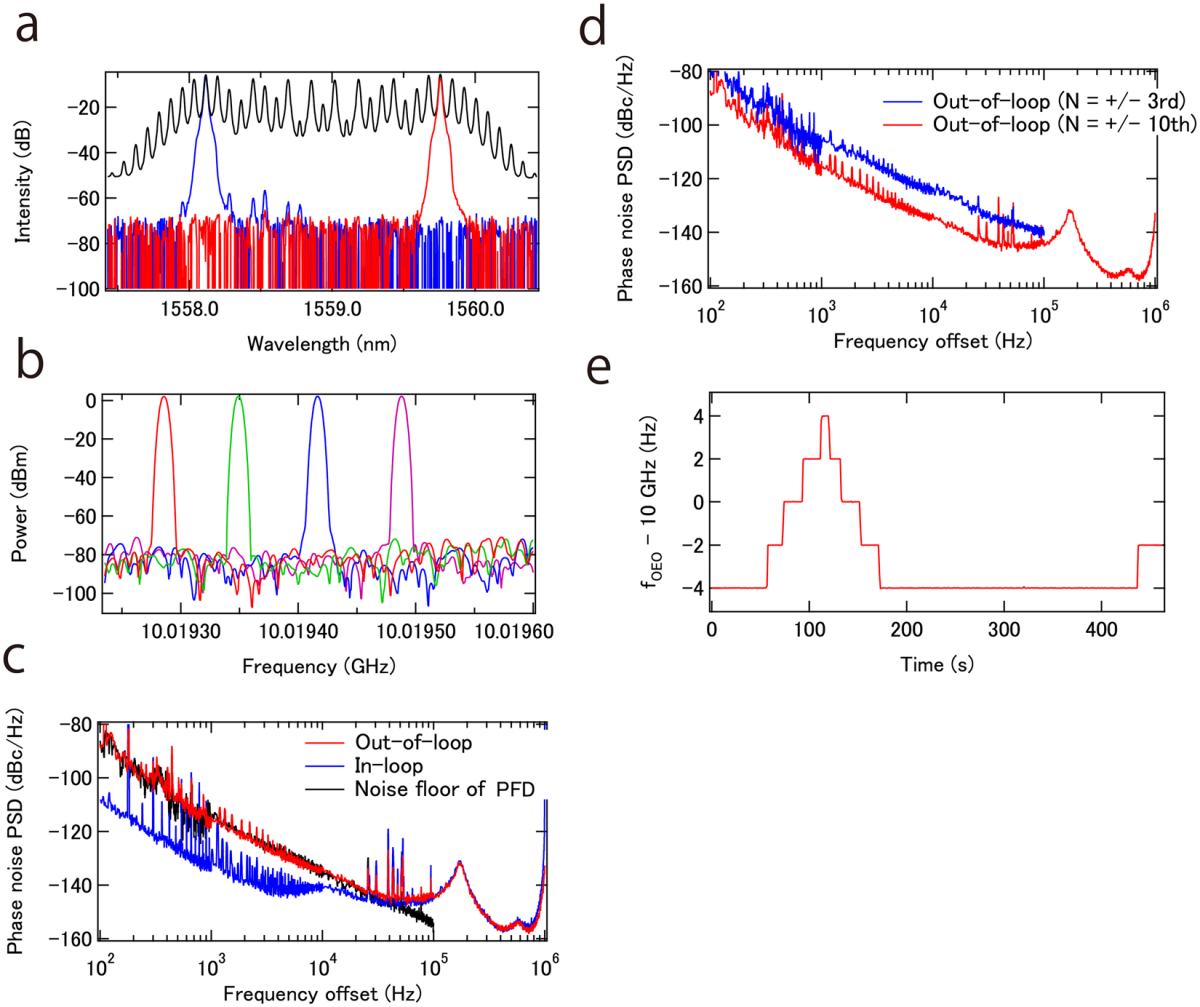
(**a**) Optical spectra of the EO comb (black) and bandpass-filtered EO comb at +10 th (red) and −10 th (blue) harmonics. (**b**) Fine tuning of the OEO oscillation frequency. (**c**) In-loop and out-of-loop phase noise of the OEO for a 10 GHz carrier. (**d**) Out-of-loop phase noise of the OEO for a 10 GHz carrier when using +/−3 rd and +/−10 th EO comb modes. (**e**) OEO oscillation frequency with locking to an external synthesizer.

When the tunable OEO is locked to the PFD, a fraction of the output from the OEO, appropriately amplified with an RF amplifier drives the phase modulator, generating an EO comb (Fig. 3a). The EO comb is then used as input to the TWDI. With locking of the OEO to the PFD, fine/continuous frequency tuning of the OEO is obtained by tuning a delay control stage in the PFD (Fig. 3b). Please also refer to a supplementary movie. The mode-hop free continuous tuning range is about 300 kHz without control of the delay stage in the tunable OEO. However, by adjusting the delay in the tunable OEO by the same amount as in the PFD, tuning by more than 1 FSR is obtained, allowing for synthesis of microwave frequencies in the whole OEO tuning range without any frequency gaps.

The in-loop and out-of-loop phase noise PSD for an OEO with 10 GHz carrier are shown in Fig. 3c. Please refer to the methods for more detail. When the +/−10 th sidemode orders of the EO comb are used, the phase noise of the OEO is suppressed by more than 50 dB, compared with that of the free-running OEO. The out-of-loop phase noise is larger than the in-loop phase noise below 50 kHz Fourier frequency offset, indicating, in this frequency range, the obtained phase noise is limited by the sensitivity of the PFD. Actually, the estimated sensitivity of the PFD well overlaps with the out-of-loop phase noise. The sensitivity limit of the PFD comes from white phase noise from either electric amplifiers after PDs in the PFD or shot noise of the PDs above 500 Hz, which is converted to 1/f^2^ phase noise for the PFD through the delayed transfer function. Below 500 Hz, 1/f phase noise is observed likely caused by electric amplifiers and PDs in the PFD, which is converted to 1/f^3^ for the PFD through the delayed transfer function. Above 50 kHz Fourier frequency offset, out-of-loop follows in-loop phase noise. In this frequency range, not the PFD, but feedback gain limits the achievable phase noise. A servo bump is clearly observed around 130 kHz. To verify that indeed the sensitivity of the PFD limits achievable phase noise, out-of-loop phase noise, with use of the +/−3 rd sidemode orders of the EO comb, was also measured. As shown in Fig. 3d, about 10 dB of excess phase noise is observed, which is due to the decrease in magnification factor, i.e. −10 dB ~ 20 × log(6/20). According to this result, use of higher order sidemodes of the EO comb lowers phase noise of the tunable OEO. Thus even larger reduction in phase noise can be achieved by using a phase modulator with ultra-low half wave voltage. Alternatively, cascading of phase modulators allows for the generation of higher order sidemodes, but at the expense of a more complicated system^34–36^. Again note that the OEO does not have any high oscillation modes up to 1 MHz. Nevertheless, the OEO exhibits very low phase noise for tunable OEOs as summarized in the discussion section.

Regarding long-term stability, the present system is limited by fiber delay fluctuations in the PFD, leading to an OEO oscillation frequency drift of about 1 kHz/5 min, likely caused by temperature fluctuation. However, if long term stability is required, the OEO oscillation frequency can be phase locked to an external reference such as a reference derived from GPS by feeding back to the fiber delay control stage in the PFD. In the experiment, the OEO oscillation frequency is phase locked to a commercial external synthesizer by feeding back to a fiber length control module based on a PZT in the PFD, allowing stable, high resolution frequency tuning as shown in Fig. 3e. The locking bandwidth should be small enough (<100 Hz) so as not to degrade the phase noise of the OEO.

## Heterodying two cw lasers

**Figure 4:**
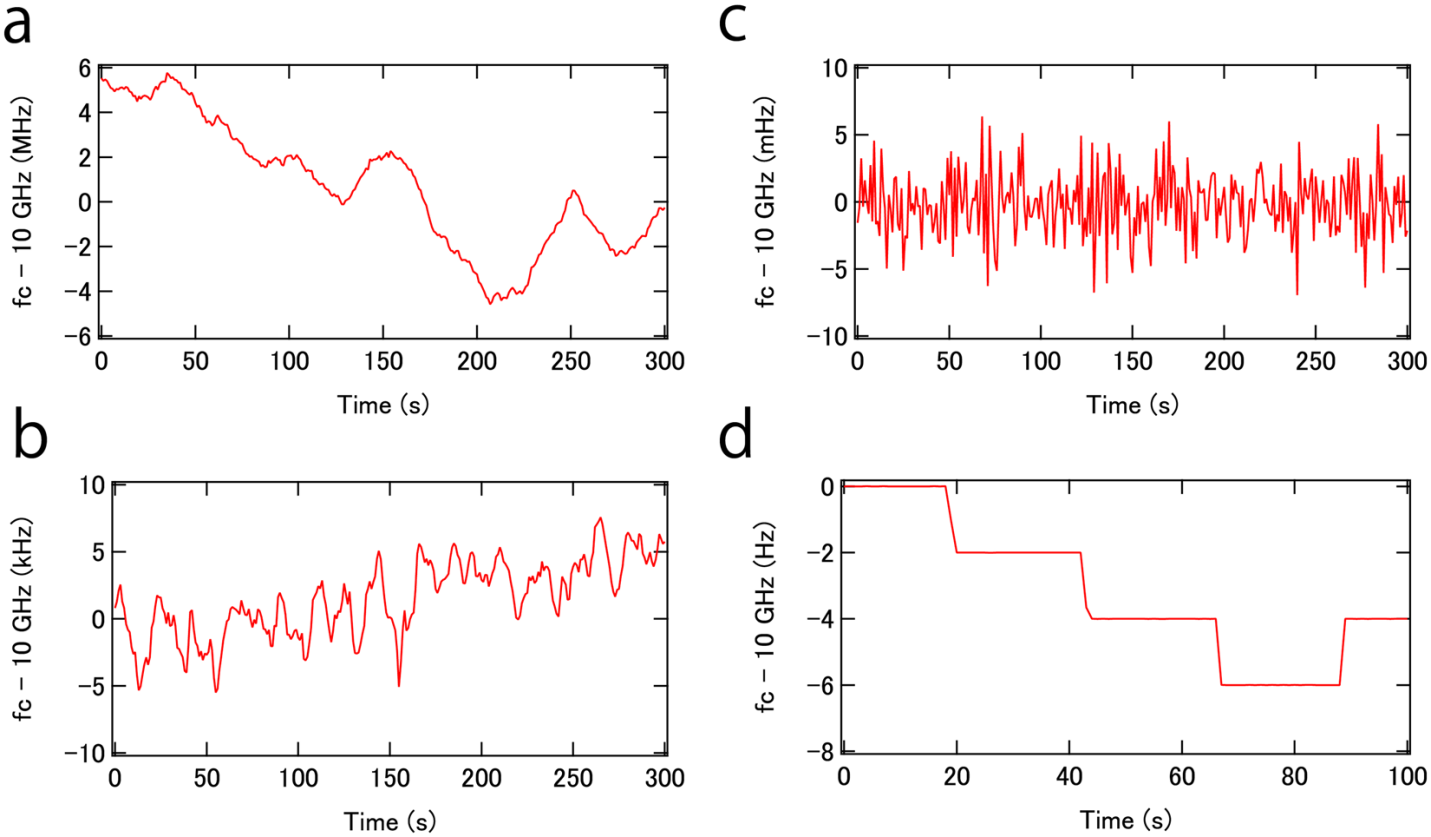
(**a**) Beat frequency between two cw lasers without locking to the PFD. (**b**) Beat frequency between two cw lasers with locking to the PFD. (**c**) Beat frequency between two cw lasers when the PFD is locked to an external reference. (**d**) Fine tuning of beat frequency between two cw lasers when the PFD is locked to an external reference.

Here a set-up as explained with respect to Fig. 1a,c is implemented, where two independent cw lasers are injected into the PFD. The frequency separation is selected between 10 and 400 GHz by changing the optical frequency of one of the two cw lasers. An optical coupler is inserted in one arm of the iMZI. The output from the coupler includes the two cw lasers. In a first experiment, the output is photodetected, generating a beat at the separation frequency. We were not able to detect beat frequencies higher than 30 GHz because of the limited bandwidth of the PD available in our lab. Figure 4a shows the frequency drift of the beat at 10 GHz carrier frequency without locking to the PFD. The beat drifts about 10 MHz in 5 minutes. With locking to the PFD by feeding back to one of the two cw lasers, the drift is suppressed by a factor of 1000 (Fig. 4b). Although the drift may not be critical for applications such as radar and wireless communication, the drift needs to be eliminated when the system is used as a synthesizer. In a demonstration, we phase-locked the beat to an external reference by feeding back to a fiber length control module based on a PZT in the PFD, similar to what was used in the experiment with the tunable OEO, thereby generating an in-loop frequency drift at the sub-Hz level as shown in Fig. 4c. The frequency can be adjusted by changing the reference frequency as shown in Fig. 4d with 2 Hz frequency step, which is the minimal frequency step of our external reference. Note that the feedback bandwidth of the phase locked loop should be small enough so as not to degrade the phase noise of the beat.

**Figure 5:**
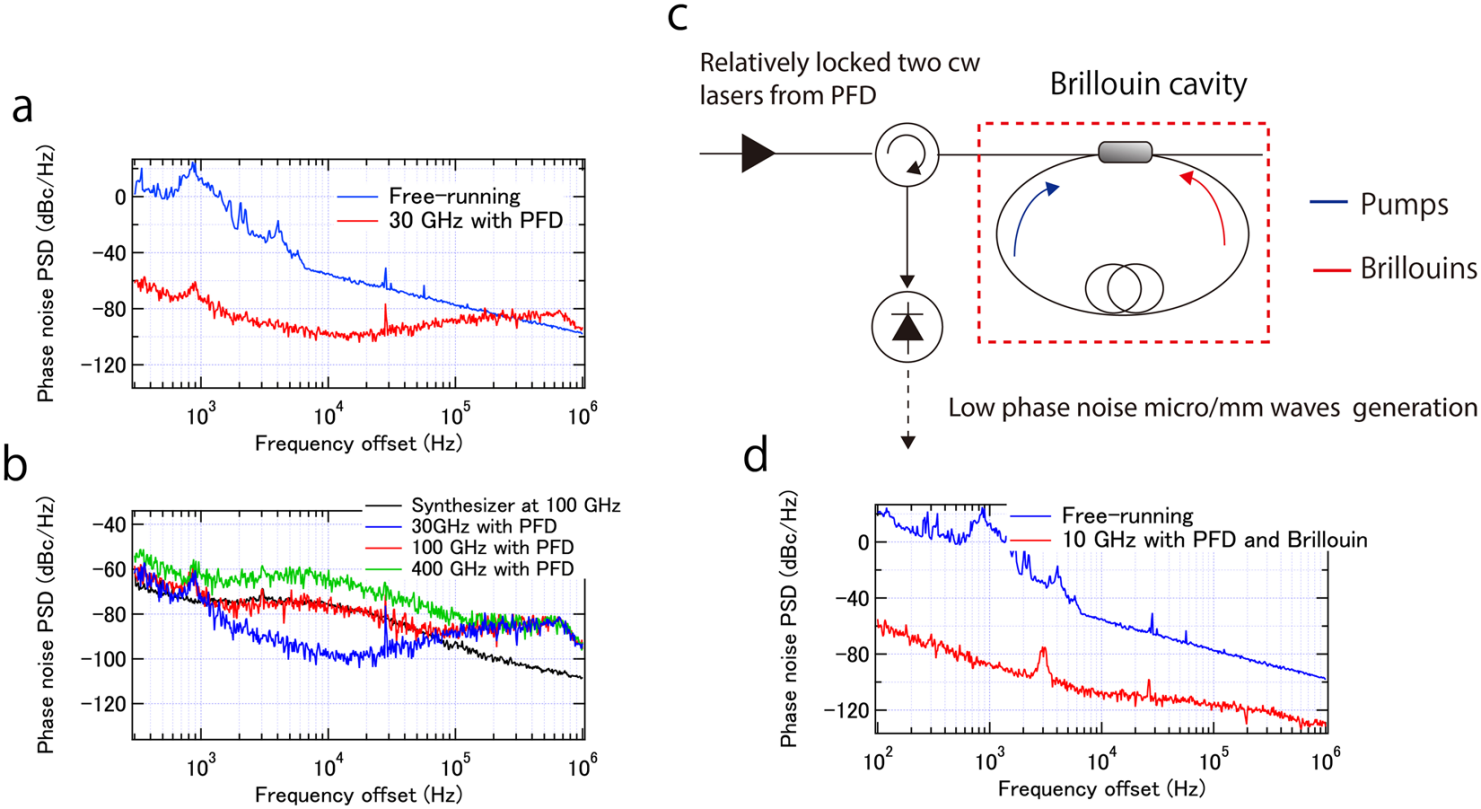
(**a**) Phase noise PSD of the beat without (blue) and with (red) locking to the PFD. (**b**) Relative phase noise PSD between two cw lasers at 30 GHz (blue), 100 GHz (red) and 400 GHz (green). Phase noise of the synthesizer scaled at 100 GHz is also shown (black). (**c**) Schematic of the Brillouin cavity. (**d**) Phase noise of the beat between two Brillouin tones (red). Free-running phase noise PSD of the beat between two cw lasers is also shown (blue).

Phase noise without locking to the PFD is equal to the relative phase noise between two cw lasers (Fig. 5a). The relative phase noise with locking to the PFD is suppressed by more than 60 dB at low frequency offsets (Fig. 5a). A servo bump is observed at about 650 kHz. The feedback bandwidth is limited by both loop length (~50 m) and the modulation bandwidth of the cw laser. Although only the phase noise for a 30 GHz heterodyne signal is shown, note that no phase noise magnification was observed in the range from 10 to 30 GHz, indicating that relative phase noise between the two cw lasers does not depend on generated carrier frequency. Therefore we believe that heterodyning of two cw lasers can be scaled to the mm/THz range without phase noise degradation.

Indeed, in the following we demonstrate that phase noise is independent of carrier frequency, specifically for frequency intervals at 100, 200 and 400 GHz. Because such high bandwidth PDs are not available in our lab, indirect phase noise measurements were carried out, in which relative phase noise between the two cw lasers is measured through an EO comb with 10 GHz comb spacing. For more detail refer to methods. The result is shown in Fig. 5b. Since the phase noise of our 10 GHz synthesizer (Hewlett-Packard, 8341A) is not low, the phase noise of the EO comb cannot be ignored, which hampers phase noise measurements below 100 kHz frequency offset. However, as shown in Fig. 5b, phase noise above 100 kHz frequency offset for 100 and 400 GHz heterodyne beats is the same as for 30 GHz (200 GHz is also the same, although not shown in Fig. 5b for simplicity). Although the measurement is indirect, we believe mm waves from high bandwidth PDs have the same phase noise, because amplitude to phase noise conversion is typically around −30 dB for UTC^37^ and PIN^38^ photodiodes. Also, the saturation current of recent UTC photodiodes with bandwidths of several hundred GHz can be more than 1 mA (e.g. UTC-PD Photomixer Module from NTT Electronic), resulting in an expected shot-noise below −150 dBc/Hz.

As shown in Fig. 5a, suppression of phase noise above 100 kHz frequency offset is limited because of the limited feedback bandwidth. To overcome this, a Brillouin cavity^39,40^ can be optionally installed. The experimental setup is shown in Fig. 5c. The output from the coupler in the one arm of the iMZI, which includes the two correlated cw lasers, is coupled into the Brillouin cavity to excite stimulated Brillouin scattering. More detail is shown in the method section. Because of the common mode cavity, the narrow Brillouin gain bandwidth, and cavity dynamics, phase noise especially at high frequency offsets can be effectively suppressed as shown in Fig. 5d. We also confirmed phase noise is the same for 10, 20, and 30 GHz. Overall, by employing both the PFD and a Brillouin cavity, phase noise over a broad frequency offset range can be effectively suppressed. Further phase noise reduction is feasible by using intrinsically low phase noise cw lasers (e.g. Sub-Hz Linewidth Semiconductor Laser from OEwaves), because the obtained phase noise is likely limited by the phase noise reduction factor of the Brillouin cavity^40^.

## Discussion

We compared the obtained phase noise of our precision tunable OEO with other frequency tunable synthesizers, including other types of tunable OEOs^19–21,23^, systems based on optical frequency combs^7^, and commercial frequency synthesizers based on RF technology (e.g. N5183B with low phase noise option from Keysight Technologies) (Table 1). Although exceptional performance is demonstrated by using optical frequency combs especially at low frequency offset, the performance relies on ultra-stable optical reference cavities, which are hard to operate, complex, and bulky. Such systems are very useful for specialized metrology labs, but are not adequate for the real world. This is because more practical methods, i.e. tunable OEOs have been developed, which sacrifice phase noise performance in favor of simplicity and cost. When comparing various tunable OEOs, not only phase noise, but also the higher oscillation mode frequencies should be discussed. Simply incorporating longer fiber delays in OEOs produces lower phase noise, but the frequency of the first higher oscillation mode becomes correspondingly smaller. From this point of view, our tunable OEO shows the best performance, i.e. low phase noise while pushing out the first higher oscillation mode to 1 MHz. Finally, we compared our tunable OEO with commercial frequency synthesizers. Although at first glance our tunable OEO performs only marginally better when looking at Table 1, our tunable OEO shows 20 dB better performance above 100 kHz frequency offset. More importantly, our tunable OEO has significant benefits, namely no principle phase noise degradation with scaling of carrier frequency. Though our demonstration here was limited to the range from 6 GHz to 18 GHz, the system can be extended to higher carrier frequencies. The PFD for the tunable OEO can be easily upgraded by using a larger bandwidth phase modulator. The tunable OEO will require a high bandwidth intensity modulator, photo detector, RF amplifier, and YIG filter. Fortunately, all these are commercially available at least up to 50 GHz.

**Table 1:** Phase noise of various synthesizers for 10 GHz carrier*.

Source	100 Hz	1 kHz	10 kHz	100 kHz	500 kHz	f_high_osc_^****^	Phase noise multiplication	Memo
OFD (comb)^7^	−120**	−130	−138	−140	−143	No	Yes	Require an ultra-stable optical reference.
PS-FBG (OEO)^19^	−45	−70	−100	−125	None***	450 kHz	No	
Brillouin (OEO)^20^	−50	−75	−90	None	None	100 kHz	No	
PM + OBPF (OEO)^21^	−60	−90	−120	−135	None	130 kHz	No	
Conventional PFD (OEO)^23^	No data	−105	−136	None	None	28 kHz	No	Although higher oscillation modes are partially suppressed, the system requires many high bandwidth components.
**Present work (OEO)**	**−89**	**−115**	**−135**	**−143**	**−155**	**1 MHz**	**No**	
RF synthesizer*****	−90	−118	−130	−125	−122	No	Yes	

*25 GHz for PM + OBPF. **Unit of phase noise is dBc/Hz. ***We use “None” for the offset frequencies higher than f_high_osc_. ****f_high_osc_ stands for frequency of first high oscillation mode. *****N5183B with low phase noise option from Keysight Technologies.

Regarding the phase noise performance achieved with heterodyning of two cw lasers, we compare our results with other methods, which can be extended to mm waves (Table 2). These methods are based on EO combs^16,41^, optical phase locked loop (OPLL) via mode-locked combs^14^, and Brillouin cavities^42^. Note that the method based on EO combs cannot be easily extended beyond 300 GHz because of the requirement for many EO comb modes. In addition, the performance is limited by phase noise from the RF synthesizer required for generation of the EO comb. OPLL do not exhibit phase noise degradation with carrier frequency, but the achievable phase noise is limited by shot noise (−90 dBc/Hz). Since the reported phase noise data from OPLL are 10 years old, the data were updated. Please see the supplementary material. State-of-the art phase noise for mm wave generation is reported in ref.^42^. The method is based on a Brillouin cavity similar to the present system. By using two phase locked loops to suppress multimode excitation of Brillouin scattering, the use of a Brillouin cavity with a long fiber (~110 m) was enabled, resulting in low phase noise. However, that method requires two low phase noise RF synthesizers around 10 GHz and two narrow optical bandpass filters. In our system, by pre-stabilizing two cw lasers via the PFD, the relative phase noise of the output from our Brillouin cavity shows as low a phase noise as ref.^42^ despite use of a shorter fiber (20 m) without requirement for low phase noise synthesizers. In addition, the PFD for heterodyning two cw lasers can be simplified. In the demonstration, we used two optical BPFs to separate the two cw lasers. However, a bi-directional configuration can be implemented instead of two optical BPFs. Please see the supplementary material. In such a configuration, the two cw lasers are input to an iMZI from opposite directions. Phase noise of the two cw lasers is then photo detected on opposite sides of the iMZI. To prevent the injection of one cw laser to the other, isolators need to be included. Once phase noise of the two cw lasers is detected independently at the PDs, the signals are mixed to generate an error signal to lock the two cw lasers in the same way as demonstrated here. The configuration is easier to implement for optical frequency tuning of one of the two cw lasers because no tuning of the bandpass filters is required. Finally, we like to comment on mm-wave generation based on RF technology. Recent progress enables carrier frequency multiplication even up to THz, starting from a 10–20 GHz source (e.g. Millimeter-Wave Accessories from Keysight Technologies). However, phase noise degradation is unavoidable. In addition, because the bandwidth of frequency multipliers is limited, having a broad tunable frequency range with one frequency multiplier is difficult.

**Table 2:** Phase noise of various methods for mm wave generation.

Source*	100 Hz	1 kHz	10 kHz	100 kHz	1 MHz	Phase noise multiplication	Memo
EO comb^41^	−50	−88	−92	−92	−110	Yes	
OPLL (ours, internal)	−80	−90	−90	−73	−95	No	
Brillouin cavity^42^	−62	−90	−105	−100	−125	No	Require two low phase noise synthesizers around 10 GHz
**Present work**	**−60**	**−90**	**−110**	**−115**	**−130**	**No**	

^*^Carrier frequency for EO comb is 125 GHz. Carrier frequency for OPLL and present work is up to 30 GHz. Carrier frequency for Brillouin cavity is measured at 10 GHz.

## Conclusion

We propose and demonstrate a novel ultra-high sensitivity photonic frequency discriminator, which can serve as a universal tool for low phase noise micro and mm-THz wave synthesis. Based on frequency locking to the PFD, low phase noise microwave signals are generated from a tunable OEO, whereas low phase noise mm wave signals can be obtained from heterodyning two cw lasers. In a proof-of-concept, low phase noise frequency synthesis from an OEO continuously tunable from 6 GHz to 18 GHz with a frequency resolution of 2 Hz is demonstrated. Unlike for conventional low phase noise OEOs, short fiber loop lengths are permissible without excessive phase noise degradation because of the superior sensitivity of the novel PFD, which in turn pushes the high oscillation mode frequency out to 1 MHz. For heterodyning of two cw lasers, the presented phase noise measurements imply no carrier frequency dependent phase noise degradation up to carrier frequencies of at least 400 GHz. Appropriate utilization of the PFD as presented here facilitates a significant improvement over the phase noise performance limits of conventional RF technology within a common and simple photonic architecture, applicable not only to microwaves, but also mm and – THz waves. The present system even rivals or improves on the performance of existing photonic methods (as listed in Tables 1 and 2, developed for limited frequency ranges). It should also be pointed out that further improvements in performance compared to what is presented here are possible, e.g. by using a phase modulator with ultra-low half wave voltage or cascading phase modulators for the tunable OEO and using intrinsically low phase noise cw lasers for heterodyning as discussed before. The demonstrated PFD offers a new paradigm for future low phase noise precision frequency synthesizers up to the THz frequency range, with superior performance and enhanced utility compared to any other present day technology.

## Methods

### Photonic frequency discriminator

Two optical inputs are coupled to an iMZI through a 2 × 2, 50:50 optical coupler. Although, in principle, one AOM is enough to observe beat signals at the PDs, two AOMs (about 80 MHz and −80 MHz frequency shift) are installed to reduce detrimental effects from optical interference between the 0 th and 1 st order diffraction modes. Since the long fiber delay is non polarization-maintaining fiber, a polarization controller and polarizer are installed after the long fiber. Other than the fiber delay, all components are polarization-maintaining. One arm has a 90:10 optical coupler. The 10% output from the optical coupler is used for the experiment on locking of two cw lasers as described in the main manuscript. The output from the two arms is interfered via a second 2 × 2, 50:50 optical coupler. The iMZI is inserted into an aluminum enclosure with 4 mm thick walls. The two optical bandpass filters are tunable over a large frequency range and also have a tunable bandwidth down to a minimum bandwidth of 10 GHz. The two PDs have 2 GHz bandwidth. The signals after the PDs are amplified and mixed in an RF mixer, generating a DC signal.

### Tunable OEO

A single-longitudinal-mode cw laser is intensity-modulated by an intensity modulator and propagates through a 200 m fiber delay. The required opto-electronic conversion is obtained in a photo detector (3 dB bandwidth of 17 GHz), generating an RF signal that is amplified (frequency range of 5–20 GHz, and 35 dB gain in total), bandpass-filtered and applied to the intensity modulator. The RF bandpass filter is based on a YIG filter (3 dB bandwidth of 40 MHz and tunability from 4 to 26.5 GHz). Upstream of the intensity modulator, 10% of the RF signal is coupled out by an RF coupler, and injected to the PFD after amplification up to about 33 dBm. Low phase noise RF output is partially taken out after the amplification. Note that although we didn’t observe any excess phase noise from the amplification, even if there is excess phase noise, the excess phase noise is also suppressed, because the feedback loop tries to minimize the phase noise after the amplification. The EO comb after the phase modulator shown in Fig. 1b is split in two. One is used for phase noise suppression as shown in the main manuscript, and the other (called output 2) is used for out-of-loop phase noise characterization. Output 2 is inserted into another PFD (called PFD 2), which comprises again an iMZI, followed by optical bandpass filters, PDs, and an RF mixer as described in the main manuscript. However, for phase noise analysis the fiber lengths in the iMZI are changed. Fiber lengths of about 1 km and 100 m are chosen for phase noise measurements in the 100 Hz –100 kHz and 100 kHz and 1 MHz frequency offset ranges, respectively.

### Relative phase noise measurement between two cw lasers with frequency separation of >100 GHz

An output from one arm of the iMZI goes through a phase modulator. The phase modulator is driven by a 10 GHz synthesizer with an RF amplifier, generating sidemodes from the two cw lasers. When the frequency separation is about 100 GHz, a beat with less than 1 GHz carrier frequency between the + and −5 th sidemode orders from the two cw lasers is observed at a photo detector after optically bandpass filtering only the two sidemodes. In the same way, +/−10 th and +/−20 th orders are used for 200 GHz and 400 GHz frequency separation. Two phase modulators are used for 400 GHz frequency separation. The phase noise PSD of the beat (L_beat_(f)) can be expressed as, L_beat_(f) = L_cw1−2_(f) + (2N)^2^L_ref_(f). Here, L_cw1−2_(f) and L_ref_(f) are the relative phase noise PSD between two cw lasers and the phase noise PSD of the synthesizer. N is the order of the used sidemodes. Because of the magnification factor of (2N)^2^ for the 10 GHz synthesizer, L_cw1−2(f)_ can only be estimated from about 100 kHz to 1 MHz as shown in Fig. 5b. (2N)^2^L_ref_(f) limits the measurement below 100 kHz frequency offset. Please see the supplementary material to see experimental setup.

### Control circuit for feedback

When OEO or heterodyning two cw lasers is locked to the PFD, an output from the PFD is used as an error signal. The error signal is put into a home-made PI2D loop filter (similar to D2–125, Vescent PHOTONICS). An output from the PI2D loop filter is split to two. One goes to a fast modulator, i.e. an EOM for OEO and laser current for heterodyning two cw lasers. The other is put in a home-made integrator. The output is used for slow, but large tuning range modulator, i.e. YIG filter for OEO and PZT in the cw laser for heterodyning two cw lasers. For OEO, a home-made current buffer with an adder (ADA4870, Analog devices) is inserted between the integrator and YIG filter, because YIG filter requires high current. For OEO, a first high oscillation mode at 1 MHz needs to be suppressed in the error signal to avoid oscillation of the feedback loop at the frequency, which limits feedback gain of the feedback loop. For this, OEO loop length is set to roughly equal to the fiber length of the PFD, making the first high oscillation frequency equal to the null frequency of the PFD (Fig. S1 in the supplementary material). The first high oscillation is significantly reduced by the null frequency at the error signal.

### PFD with a Brillouin cavity

The Brillouin cavity consists of a 2 × 2 optical coupler with 90:10 coupling ratio and about 20 m fiber. The Brillouin cavity is enclosed by a 4 mm thickness aluminum box. By coupling sufficient optical power to the Brillouin cavity (~15 mW for each cw laser), back scattered Brillouin tones with frequencies of ν_cw1_ − f_Bri_ and ν_cw2_ − f_Bri_ are generated. Here, f_Bri_ is the Brillouin frequency shift. To resonantly couple two cw lasers into the Brillouin cavity, two Pound-Drever-Hall locks (PDHs) are implemented for each cw laser. To observe error signals for the PDHs, the two cw lasers are phase modulated, where transmitted light from the Brillouin cavity is photo-detected, followed by demodulation, resulting in error signals. The error signals are fedback to one of the cw lasers and a fiber length control module based on a PZT in the Brillouin cavity for the two PDHs. Please see the supplementary material for the experimental setup.

## Electronic supplementary material

Supplementary Information
