# Supplementary material for: A photonic frequency discriminator based on a two wavelength delayed self-heterodyne interferometer for low phase noise tunable micro/mm wave synthesis
Source: Sci Rep. 2018 Sep 12;8:13719. doi: 10.1038/s41598-018-31712-y (PMC6135791; doi:10.1038/s41598-018-31712-y)
Supplement: Supplementary file 1 — Supplementary Information [file 41598_2018_31712_MOESM1_ESM.docx]

**Supplementary Information**

**A photonic frequency discriminator based on a two wavelength delayed self-heterodyne interferometer for low phase noise tunable micro/mm wave synthesis**

Naoya Kuse^1*^, and Martin E. Fermann^2^

*^1^ IMRA America Inc., Boulder Research Labs, 1551 South Sunset St, Suite C, Longmont, CO, 80501, USA*

*^2^ IMRA America Inc., 1044 Woodridge Ave, Ann Arbor, MI, 48105, USA*

*e-mail: [nkuse@imra.com](mailto:nkuse@imra.com)

**1. Delay transfer function**

The delay transfer function (H(jf)) of an imbalanced Mach-Zehnder interferometer (iMZI) is expressed as,

$\left| H(jf) \right|^{2}=4\text{sin}^{2}\left( \pi\tau f \right)$ (S1).

Here, τ and f are time delay for the iMZI and the offset frequency from the carrier frequency, respectively. The delay transfer function has a null frequencies at 1/τ as shown in Fig. S1. The null frequencies for e.g. 100 m and 1 km fiber are 200 kHz and 2 MHz, respectively. The usable frequency offset is limited by the null frequency.


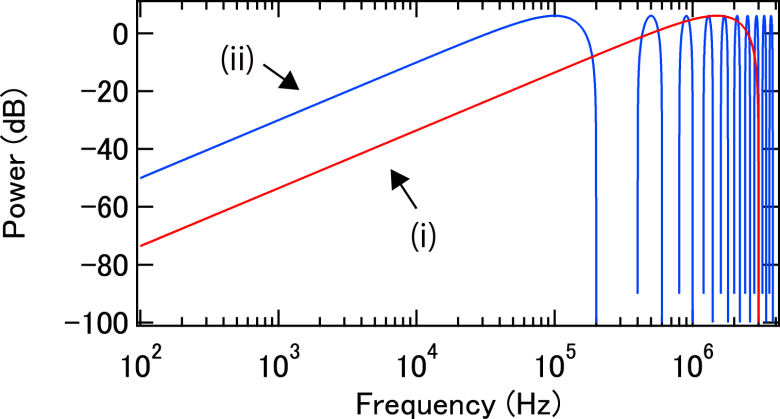


Figure S1. Delay transfer function ($\left|H(jf)\right|^{2}$) for 100 m (i, red) and 1 km (ii, blue).

**2. Relative phase noise between two cw lasers via optical phase locked loop (OPLL)**

Since reported phase noise by OPLL is 10 years old and not up to date, we updated the phase noise achievable by OPLL. A schematic of experimental setup is shown in Fig. S2a. Two cw lasers are phase locked to a mode-locked frequency comb, and out-of-loop phase noise of a beat between the two cw lasers is measured (Fig. S2b). The result is highlighted in Table 2 in the main manuscript.


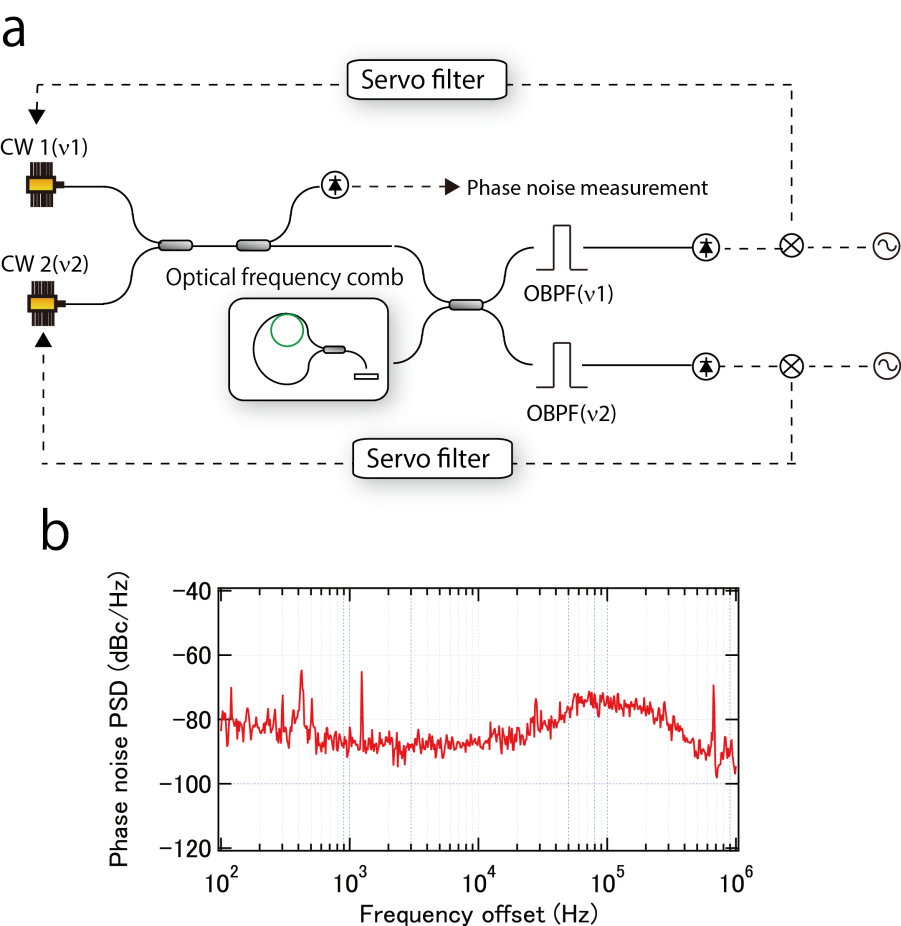


Figure S2. (a) Schematic of the experimental setup for OPLL. (b) Relative phase noise of the two cw lasers with locking to the optical frequency comb.

**3. Bi-directional setup for the photonic discriminator for locking of two cw lasers**

Although a one-directional configuration is used for locking of two cw lasers (i.e. in the same way as for phase noise reduction of a tunable OEO), a bi-directional configuration (Fig. S3) can be utilized for locking of two cw lasers. A bi-directional configuration avoids the need for two optical bandpass filters. In such a configuration, the two cw lasers are input to an unbalanced MZI from opposite directions. Phase noise of the two cw lasers is then photo detected on opposite sides of the unbalanced MZI. To prevent the injection of one cw laser to the other, isolators need to be included. Once phase noise of the two cw lasers is detected independently at the PDs, the signals are mixed to generate an error signal to lock the two cw lasers in the same way as demonstrated here.


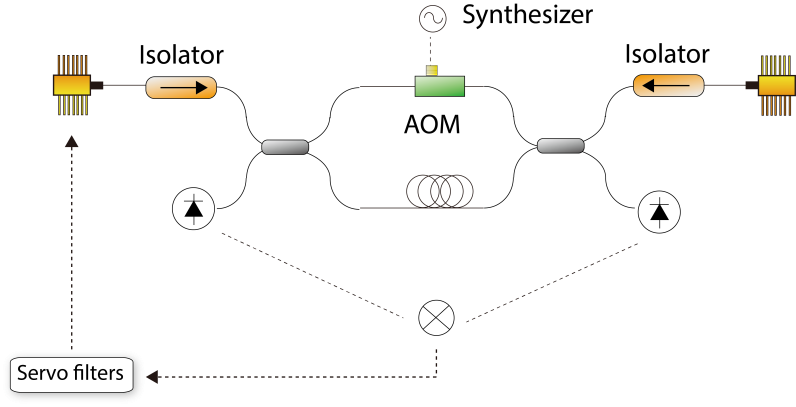


Figure S3. Schematic of the bi-directional configuration.

**4. Indirect relative phase noise measurement between two cw lasers beyond PD bandwidth**

Because the bandwidth of the PDs in our lab is limited to 30 GHz, an indirect relative phase noise measurement between two cw lasers is implemented when the frequency separation of the two cw lasers is beyond the PD bandwidth. The method uses sidemodes from the two cw lasers, which are generated by phase modulation. A schematic of the measurement is shown in Fig. S4.


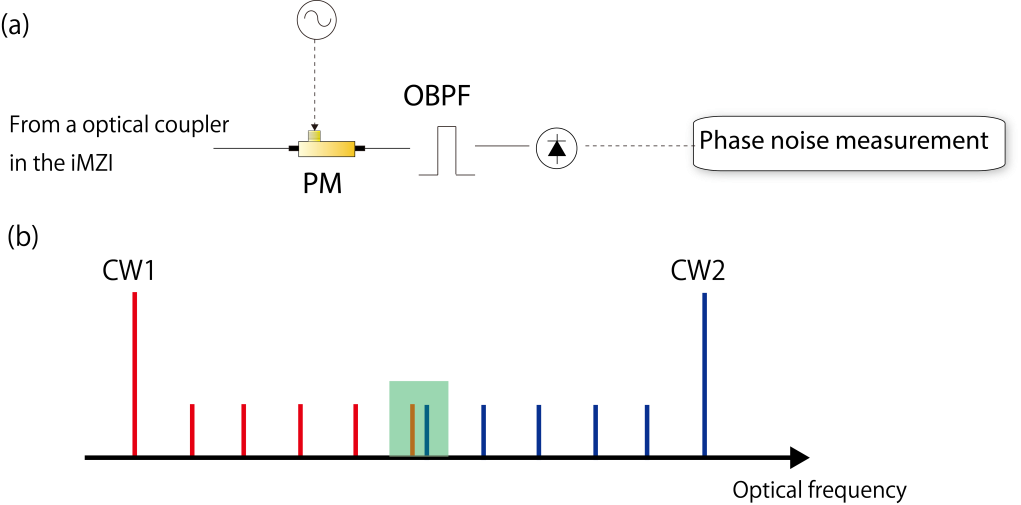


Figure S4. (a) Schematic of the experimental setup. PM, phase modulator; OBPF, optical bandpass filter. (b) Concept of the method in the optical frequency domain. Green-square shows two EO comb lines, which are taken out by the optical bandpass filter.

**5. Photonic frequency discriminator with a Brillouin cavity**

A schematic of an experimental setup for use of the photonic frequency discriminator with a Brillouin cavity for heterodyning of two cw lasers is shown in Fig. S5.


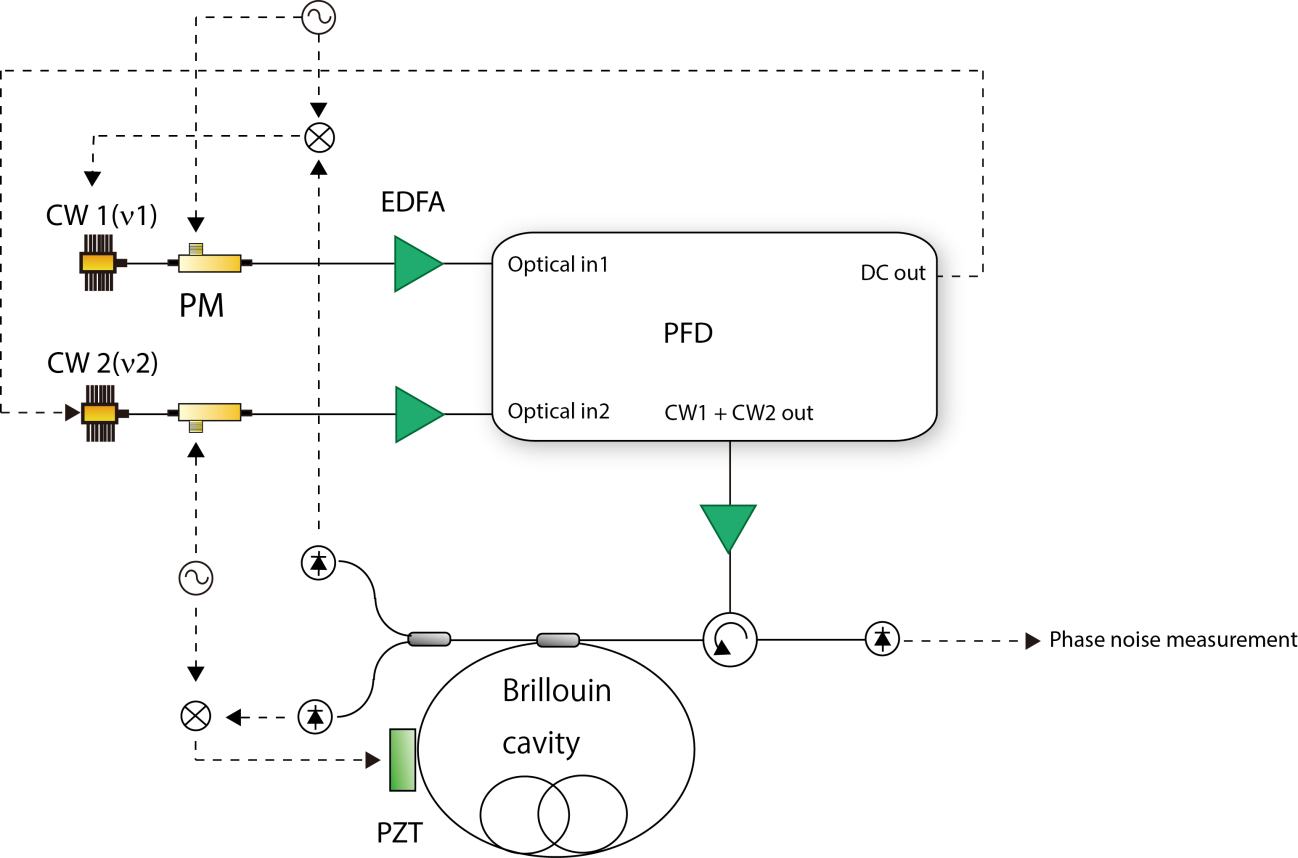


Figure S5. Schematic of the experimental setup.
